# Simulation tools for assessment of tick suppression treatments of *Rhipicephalus* (*Boophilus*) *microplus* on non-lactating dairy cattle in Puerto Rico

**DOI:** 10.1186/s13071-019-3443-6

**Published:** 2019-04-27

**Authors:** Hsiao-Hsuan Wang, Pete D. Teel, William E. Grant, Fred Soltero, José Urdaz, Alejandro E. Pérez Ramírez, Robert J. Miller, Adalberto A. Pérez de León

**Affiliations:** 1 Department of Wildlife and Fisheries Sciences, Texas A&M University, College Station, TX 77843 USA; 2 Department of Entomology, Texas A&M AgriLife Research, College Station, TX 77843 USA; 3 United States Department of Agriculture-Animal and Plant Health Inspection Service, Veterinary Services, 654 Munoz Rivera Ave. Plaza Bldg. Suite 700, San Juan, 00918 Puerto Rico; 4 United States Department of Agriculture-Animal and Plant Health Inspection Service, Veterinary Services, 2150 Centre Ave. Bldg. B, MS-3E13, Ft. Collins, CO 80526 USA; 5 Veterinary Services and Animal Health, Puerto Rico Department of Agriculture, P.O. Box 10163, San Juan, 00908-1163 Puerto Rico; 6 Cattle Fever Tick Research Laboratory, United States Department of Agriculture-Agricultural Research Service, Edinburg, TX 78541 USA; 7 Knipling-Bushland U.S. Livestock Insects Research Laboratory, and Veterinary Pest Genomics Center, United States Department of Agriculture-Agricultural Research Service, Kerrville, TX 78028 USA

**Keywords:** Agent-based, Individual-based, Modeling, Spatially-explicit, Stochastic, Integrated tick management research, *Rhipicephalus microplus*

## Abstract

**Background:**

The southern cattle fever tick (SCFT), *Rhipicephalus* (*Boophilus*) *microplus*, remains endemic in Puerto Rico. Systematic treatment programmes greatly reduced and even eradicated temporarily this tick from the island. However, a systemic treatment programme that includes integrated management practices for livestock against SCFT remains to be established in the island. We describe a spatially-explicit, individual-based model that simulates climate–livestock–SCFT–landscape interactions. This model was developed as an investigative tool to aid in a research project on integrated management of the SCFT that took place in Puerto Rico between 2014 and 2017. We used the model to assess the efficacy of tick suppression and probability of tick elimination when applying safer acaricides at 3-week intervals to different proportions of a herd of non-lactating dairy cattle.

**Results:**

Probabilities of eliminating host-seeking larvae from the simulated system decreased from ≈ 1 to ≈ 0 as the percentage of cattle treated decreased from 65 to 45, with elimination probabilities ≈ 1 at higher treatment percentages and ≈ 0 at lower treatment percentages. For treatment percentages between 65% and 45%, a more rapid decline in elimination probabilities was predicted by the version of the model that produced higher densities of host-seeking larvae. Number of weeks after the first acaricide application to elimination of host-seeking larvae was variable among replicate simulations within treatment percentages, with within-treatment variation increasing markedly at treatment percentages ≤ 65. Number of weeks after first application to elimination generally varied between 30 and 40 weeks for those treatment percentages with elimination probabilities ≈ 1.

**Conclusions:**

Explicit simulation of the spatial and temporal dynamics of off-host (host-seeking) larvae in response to control methods should be an essential element of research that involves the evaluation of integrated SCFT management programmes. This approach could provide the basis to evaluate novel control technologies and to develop protocols for their cost-effective use with other treatment methods.

**Electronic supplementary material:**

The online version of this article (10.1186/s13071-019-3443-6) contains supplementary material, which is available to authorized users.

## Background

The dairy industry on the Caribbean island of Puerto Rico is recognized as the single-largest sector of the agricultural economy, valued at over $200M annually and representing one-quarter of the island’s agricultural income [1]. The mean annual milk cow population has been estimated at 77,000 head [1]. A significant impediment to optimizing animal health and production in dairy systems is the southern cattle fever tick (SCFT), *Rhipicephalus* (*Boophilus*) *microplus*. Tick parasitism directly impairs animal growth, reproductive potential, and productivity through blood loss and irritation and indirectly through transmission of pathogens responsible for tick-borne bovine diseases, specifically bovine anaplasmosis and babesiosis [2, 3]. Losses in 2000 due to *R. microplus* infestations, bovine anaplasmosis, and bovine babesiosis were estimated at US $6.7 million [4]. Both production losses and costs of control threaten the economic stability of these enterprises.

In Puerto Rico, SCFT is the principal vector of *Anaplasma marginale*, the causal agent of bovine anaplasmosis, and *Babesia bovis* and *Babesia bigemina*, the causal agents of bovine babesiosis [2, 5]. In 1936 an eradication programme based on systematic acaricide treatments was established and by the 1950s the island was declared SCFT-free in Puerto Rico [6, 7]. SCFT was detected again in the late 1970’s, and in 1979, the USDA, Animal and Plant Health Inspection Service, Veterinary Services, and the Department of Agriculture, Commonwealth Government of Puerto Rico began a large-scale cattle fever tick eradication programme. During a 4-year period in the 1980’s approximately US $44 million were spent without noticeable reductions in tick infestations [8]. This situation was attributed to quarantine violations and feeding of grass containing ticks [7, 8], a general reluctance of farmers to accept the programme [9], and the fact that a large number of producers kept non-lactating dairy cows at different premises than their milking cows, which were outside treatment zones [7]. Industry and producer dedication, programme compliance on animal movement and strict adherence to treatment protocols are all necessary for the success of SCFT eradication programmes [9, 10]. Until recently, funding for the eradication programme was provided by the Puerto Rico Dairy Association on a voluntary basis by supplying amitraz, a formamidine acaricide, to producers. However, acaricidal products containing amitraz to treat cattle are not commercially available now. The invasive SCFT remains established in Puerto Rico and other islands of the Caribbean [11].

The presence of cattle is the single most important factor associated with the presence of southern cattle tick larvae in the Puerto Rican environment [12]. More than 95% of Puerto Rican dairy farmers in a recent survey indicated they apply acaricides for tick control; however, these treatments varied in application method and frequency, ranging from weekly to more than 40-day intervals [13]. Variation in treatment efficacy due to product selection, proportion of animals treated, treatment coverage and duration, as well as operational interruptions in treatment schedules contribute to sustaining populations of *R. microplus* and increase the risk for the development of acaricide resistance. The persistence of tick larvae in pastures used to graze and support dairy cattle is responsible for sustaining the force of tick dispersal, re-infestation and transmission of tick-borne diseases [12]. Unlike the parasitic life stages, the survival of off-host larvae is influenced directly by seasonally variable and often unpredictable environmental conditions, which differ between regions and habitats [14].

Modern dairies require management of calves, heifers, bred heifers, non-lactating cows, mature lactating cows, and bulls in an integrated system [15]. Depending on facilities, land, and infrastructure, each interaction in the system can provide biosecurity points subject to tick introduction and dispersal. For most dairies, the management of non-lactating cows and heifers is typically focused on forage pastures [16], an environment where habitats can sustain populations of *R. microplus* ticks in Puerto Rico [17], and a continuous source of tick infestation to livestock herds.

Collaborative research to develop an Integrated Tick Management Programme was supported since 2013 by the Puerto Rico Dairy Industry and the Puerto Rico Department of Agriculture in collaboration with the United States Department of Agriculture, Agricultural Research Service (USDA-ARS), and Animal Plant Health and Inspection Service-Veterinary Services (APHIS-VS). The objective of this research partnership was to test the benefits of combining technologies for sustainable control of the SCFT infesting cattle herds. An important aspect was to test the integrated use of an anti-tick vaccine with safer acaricides [11, 18, 19]. As part of this effort, we report here the development of an investigative tool to aid in integrated SCFT management for pastured non-lactating dairy cattle. More specifically, a model that simulates climate–host–parasite–landscape interactions was developed and used to assess the efficacy of acaricide treatments applied at 3-week intervals to varying proportions of the non-lactating dairy herd.

Previous models tried to predict the effect of integrated efforts to manage SCFT populations including the combined use of acaricides with anti-tick vaccines for their eradication [20–24]. Knowledge from those simulation tools was adapted to parameterize several versions of the model presented here, which represent different assumptions regarding the maximum allowable tick loads on cattle. This allowed the analysis of the model with regard to sensitivity of simulated tick loads and associated densities of host-seeking larvae in the environment to the uncertainty embodied in these assumptions. The different versions of the model were then evaluated with regard to their success in producing tick loads and off-host larval densities comparable to those reported in the absence of tick control. Finally, using each of those successful versions of the model, we examine the relationship between the proportion of a non-lactating pre-parturient cows and heifers in a dairy herd treated with acaricides and the efficacy of the treatment in reducing populations of off-host larvae from the system.

## Methods

### Model description

**Fig. 1:**
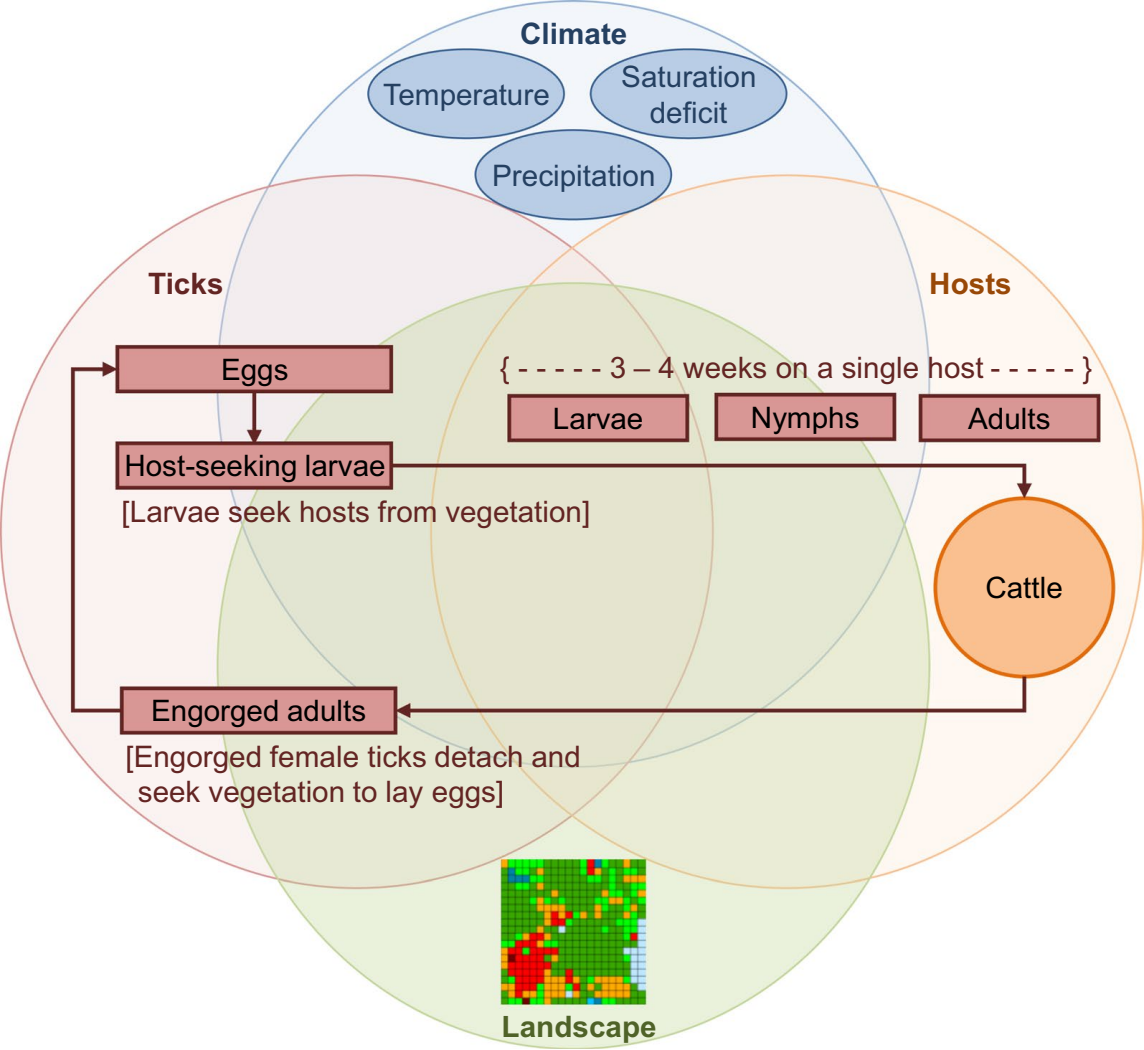
Conceptual model representing the interactions of climate variation and habitat heterogeneity on the life-cycle of the southern cattle fever tick, *Rhipicephalus* (*Boophilus*) *microplus*

The model, which is an adaptation of a model developed by Wang et al. [25] to evaluate southern cattle tick eradication methods in south Texas, USA, is spatially-explicit, individual-based, and stochastic. The model simulates the effects of climate variation, habitat heterogeneity, and acaricide applications to non-lactating dairy cattle on the life cycle of the southern cattle tick in Puerto Rico (Fig. 1). We parameterized the model to represent climatic conditions and landscape characteristics typical of dairy farms in the southwestern portion of Puerto Rico. The area is predominantly subtropical moist forest, with mean annual precipitation between 1525 and 1650 mm and a mean annual temperature of 25 °C. There is a dry season from December to April and a wet season from May to November, with minimal differences in air temperature between dry and wet seasons. Off-host larval tick abundance is higher during the wet season and is associated with areas containing > 25% woody vegetation (brushes and shrubs), and the presence of cattle [12]. In the model, climatic conditions are represented by weekly varying temperature, precipitation, and saturation deficit, and the pasture is characterized as an open meadow surrounded on three sides by woods. Off-host tick life stages include eggs, host-seeking larvae and engorged (fed) adults. On-host life stages include larvae, nymphs and adults. Acaricide effects are represented by increasing mortality rates of ticks (larvae, nymphs and adults) on individual cattle that have been treated with acaricide. We provide a detailed model description following the ODD (Overview, Design concepts and Details) protocol suggested for individual-based models by Grimm et al. [26] in Additional file 1 and a summary of model parameters and equations in Additional file 2.

### Model calibration, sensitivity analysis, and evaluation

The performance of several versions of the model parameterized to represent different assumptions regarding the maximum allowable tick loads on cattle was examined. We analyzed the sensitivity of simulated tick loads and associated densities of host-seeking larvae in the environment to the uncertainty embodied in these assumptions. Different versions of the model were then evaluated with regard to their success in producing tick loads and off-host larval densities comparable to those reported in the absence of tick control. Uncertainty regarding maximum tick loads was represented by establishing a calibration index (*k*) that limited the maximum number of tick larvae infesting an individual host at any one time, thus also imposing an upper limit on the number of nymphs and adults on any given host at any given time. An assumption in the model is that as an individual host moves about the landscape (described in Section 2.7.6 of [25]) and becomes infested with host-seeking larvae (described in Section 2.7.4 of [25]), the number of these larvae that can infest the host depends on the number of larvae already on the host. Thus, the value of k functions to establish an arbitrary upper limit, or “carrying capacity,” on the number of larvae that can be on the host at any given time. This establishes an upper limit on the number of adult ticks infesting the host given the survival rates of on-larvae, nymphs, and adults (described in Section 2.7.5 of [25]). The number of simulated adult ticks on cattle then can be compared with field data. We ran 10, 5-year, Monte Carlo simulations at each of numerous values of *k* ranging from 1 to 11000 and calculated (i) the mean loads of adult ticks on cattle during the 5th year of simulations and (ii) the mean densities of host-seeking larvae during the 12th week of the 5th year, which was representative of the annual peak densities occurring in mid-March (the different values of *k* produced no qualitative differences in seasonal patterns or year-to-year trends of either adult tick loads or off-host larval densities). To facilitate comparisons with values reported in the literature, we converted the number of simulated adult ticks on cattle to the equivalent number of standard-sized female ticks (4.5–8.0 mm in length) by multiplying by 0.5 (assuming a 50:50 sex ratio) and then by 0.36 (assuming ticks were of standard size during their 16th through 20th day on the host [27], i.e. during approximately 36% of their time as adults on the host).

### Model application

We assessed the efficacy of applying an acaricide at 3-week intervals for 9 consecutive months to different 5% intervals of the non-lactating cattle herd ranging from 100% to 45% of its population. Acaricide applications began on January 1 of the second year of each 5-year simulation, using weather data for southwestern Puerto Rico, as described in the “Model description” section above. Results from the sensitivity analysis and evaluation informed the decision to execute the experimental design using 2 versions of the model: (i) with a value of *k*, which produced the maximum load of standard-sized females per host, and (ii) with another value of *k*, which produced the maximum density of host-seeking larvae. Using each of these 2 versions of the model, we ran 10 Monte Carlo simulations of each of the acaricide application scenarios. During each simulation, we monitored the relative density of host-seeking larvae in the environment and summarized these results in terms of (i) the probability of eliminating host-seeking larvae from the system and (ii) the number of weeks after the first acaricide application to elimination. Ten Monte Carlo simulations of each scenario allowed detection of a difference of 5000 in the density of host-seeking larvae among treatments with a Type I error = 0.05 and a Type II error = 0.01, or a difference ≈ 3.1% in the overall mean number of host-seeking larvae.

## Results

### Model calibration, sensitivity analysis, and evaluation

**Fig. 2:**
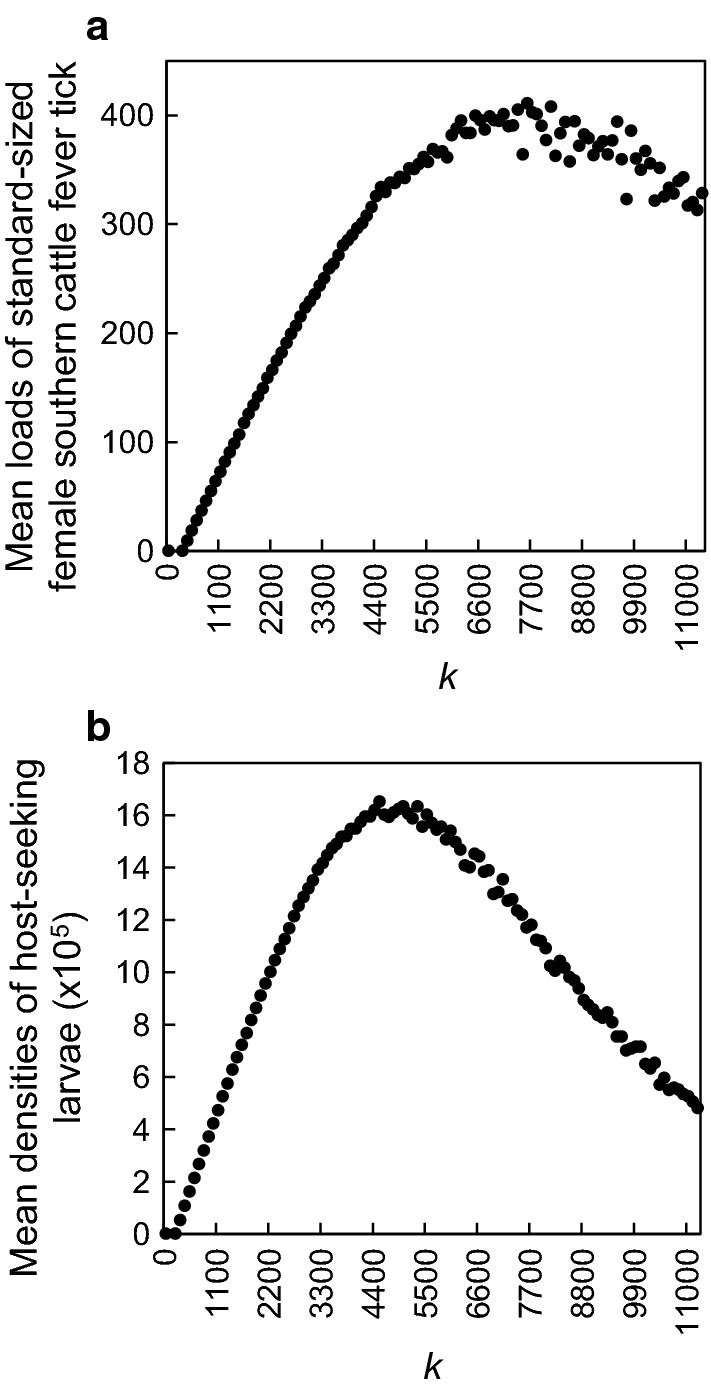
Responses of (**a**) mean loads of standard-sized female southern cattle fever tick, *Rhipicephalus* (*Boophilus*) *microplus*, on cattle and (**b**) mean densities of host-seeking larvae in the environment to changes in values of the calibration parameter *k*. Means are based on 10, 5-year, Monte Carlo simulations at each *k* value

**Fig. 3:**
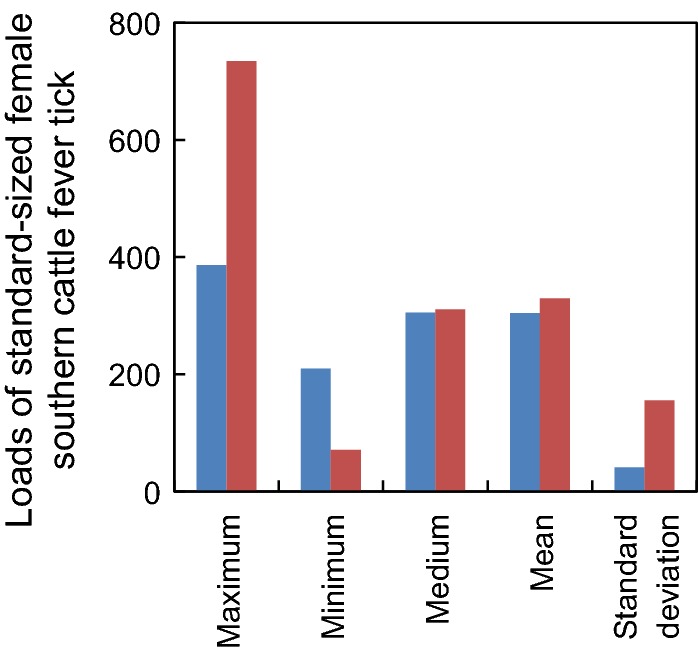
Maximum, minimum, median and mean (± standard deviation) loads of standard-sized female southern cattle fever tick, *Rhipicephalus* (*Boophilus*) *microplus*, on cattle during simulations at *k* values of 4000 (blue bars) and 11,000 (red bars). Bars represent means based on 10, 5-year, Monte Carlo simulations at each value of *k*

Results of the sensitivity analysis for the calibration index *k* indicated that as we raised the limit on the maximum allowable tick load, mean tick loads increased from 0 (*k* < 5) to ≈ 400/individuals (6500 ≤ *k* ≤ 7500) and then declined to ≈ 350/individuals (*k* = 11000) (Fig. 2a). Mean larval densities increased from ≈ 4100/ha (*k* = 10) to ≈ 1,600,000/ha (3900 ≤ *k* ≤ 5400) and then declined to ≈ 500,000/ha (*k* = 11000) (Fig. 2b). The decrease in both mean tick loads and mean larval densities at higher values of *k*, as well as the more jagged appearance of the descending portion of these curves, resulted from a shift from a relatively host-limited system to a relatively larvae-limited system (at *k* ≈ 4000). That is, for *k* values less than approximately 4000, all cattle had essentially the same probability of collecting larvae at any given time, which depended primarily on the habitat they were in and their current tick load. However, at increasingly higher *k* values an increasing number of cattle had a markedly reduced probability of collecting larvae at any given time because the local supply of larvae already had been exhausted by their peers. This is evidenced by the fact that maximum tick loads during simulations were markedly higher (≈ 700) while minimum tick loads were marked lower (≈ 75) at *k* = 11,000 compared to *k* = 4000 (≈ 400 and ≈ 200, respectively), although the median and mean tick loads were comparable (≈ 300) (Fig. 3).

Both simulated tick loads and associated densities of host-seeking larvae were sensitive to changes in the assumptions made regarding maximum allowable tick loads. However, neither increased monotonically with increasing values of *k*, and the ranges of both seemed reasonable, if perhaps a bit conservative, in view of values reported in the literature. Bourne et al. [28] reported that *Bos taurus* cattle in central and southern Queensland carried an average of 465 and 302 standard-sized female cattle fever ticks, respectively. The model developed by Mount et al. [29] predicted ≈ 600–700 standard-sized female cattle fever ticks on *B. taurus* cattle when simulating environmental conditions representative of San Juan, Puerto Rico, with the associated densities of host-seeking larvae varying seasonally from ≈ 2–5 million per hectare. Our model predicted a maximum of 411 standard-sized females per cow at *k* = 7300 and simulated loads of standard-sized females > 300 for *k* > 3800, with ≈ 1.6 million host-seeking larvae per hectare for 3900 ≤ *k* ≤ 5400. (Note that if we assume females are standard-sized during their 15th through 21st day on the host, rather than during their 16th through 20th day on the host, our model predicts a maximum of 507 standard-sized females per cow).

### Model application

**Fig. 4:**
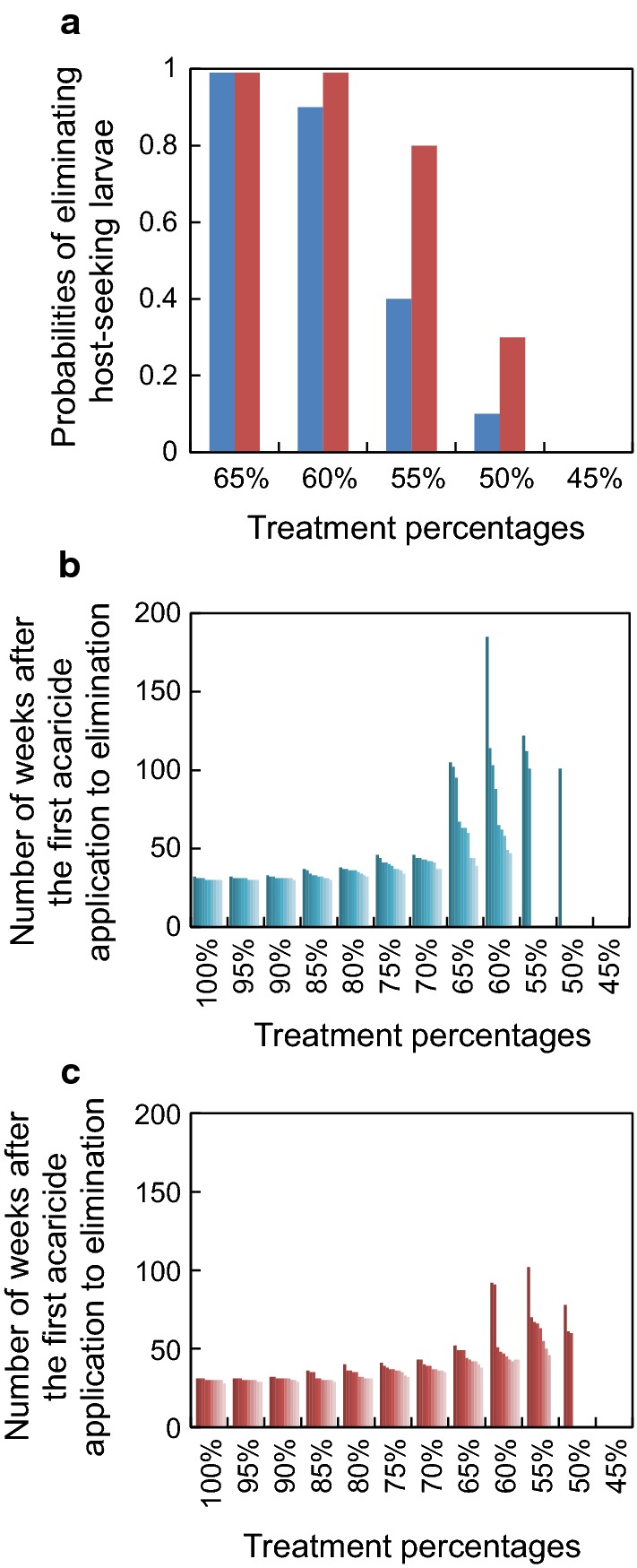
Summary of responses of simulated southern cattle fever tick, *Rhipicephalus* (*Boophilus*) *microplus*, populations to dipping cattle in acaricide every 3 weeks for 9 months when the indicated percentages of the cattle are treated. **a** Probabilities of eliminating host-seeking larvae from the simulated pasture. Blue bars and red bars represent results from the versions of the model with *k* = 4200 and *k* = 7300, respectively. Ranges in the number of weeks after the first acaricide application to elimination with *k* = 4200 (**b**) and *k* = 7300 (**c**). No bars indicated eliminations did not happen under the specific treatments. Results are based on 10, 5-year, Monte Carlo simulations of each treatment level

Of the 2 model application simulations conducted, *k* = 7300 for the version that produced the maximum load of standard-sized females per host, and *k* = 4200 in the version that produced the maximum density of host-seeking larvae. Probabilities of eliminating host-seeking larvae from the simulated pasture system decreased from ≈ 1 to ≈ 0 as the percentage of cattle treated decreased from 65 to 45, with elimination probabilities ≈ 1 at higher treatment percentages and ≈ 0 at lower treatment percentages, regardless of the version of the model used (Fig. 4a). For treatment percentages between 65% and 45%, a more rapid decline in elimination probabilities was predicted by the version of the model that produced higher densities of host-seeking larvae (*k* = 4200). The 2 versions of the model yielded similar results when the number of weeks after the first acaricide application to elimination of host-seeking larvae was variable among replicate simulations within treatment percentages, with within-treatment variation increasing markedly at treatment percentages < 65 (Fig. 4b, c). Number of weeks after first application to elimination generally varied between 30 and 40 weeks for those treatment percentages with elimination probabilities ≈ 1, regardless of the version of the model used. For those treatments in which host-seeking larvae were not eliminated, larval densities were higher in the woods bordering the meadow than in the meadow. At the end of the simulations in which 45% of the cattle were treated, mean (± SD) larval density in the woods was 19,602 ± 8387 per hectare, compared to 4392 ± 2623 per hectare in the meadow (with *k* = 7300), and 70,673 ± 21,347 compared to 26,736 ± 12,221 (with *k* = 4200).

## Discussion

Our simulations have focused on the relationship between the proportion of non-lactating dairy cattle treated with acaricides on a systematic interval and the efficacy of the treatment in eliminating populations of off-host larvae. We have confined our analyses to a control method consisting of acaricide applications every three weeks for nine consecutive months. Our results show that treating less than 65% of the herd (regardless of the version of the model used) markedly increases the probability that host-seeking larvae will not be eliminated from the system. Conversely, increasing the percentage of animals treated to more than 65% shortens the time to elimination and provides increased assurance that host-seeking larvae will be eliminated from the system.

Our simulations assumed a closed system from the standpoint of SCFT management, which is a scenario provided by enhanced farm biosecurity [30]. Under this assumption, neither cattle nor ticks enter or leave the simulated pasture. However, in operating dairy farms of Puerto Rico non-lactating cows are cycled through “dry herd” pastures we simulated, which are often infested with SCFT [5]. Typically, non-lactating cows arrive from, and return to, the milking herd facilities. Depending on the operation, they also may spend time in a pasture with bulls and/or in a pasture where dams and their newly-born calves are kept. Also, while in the “dry herd” pasture, they may be exposed to host-seeking larvae introduced by other hosts or via infested hay used to supplement forage resources. The existence of “refuge infestations” [25] maintained by alternate hosts in wooded areas within the farm may provide a continuous source of SCFT for the re-infestation of cattle. Consequently, to the extent that such “biosecurity leaks” lead to tick introduction and dispersal within the farm, the effect of acaricide treatments to manage SCFT populations will be diminished.

Models such as the one used in this study are not designed to make precise predictions, but, rather, to provide exploratory tools to investigate climate–host–parasite–landscape interactions [24, 25, 31–33]. Within the present tick control context, the goal was to provide an investigative tool to aid in the preparedness for, possible prevention of, and integrated SCFT management response to, outbreaks of bovine babesiosis and anaplasmosis in areas where the SCFT remains established like the island of Puerto Rico. Thus, it is important to communicate clearly the uncertainty that necessarily is associated with model predictions of how complex ecological systems might respond to changing environmental conditions and/or management actions [34]. Uncertainty in predictions of complex models results from assumptions made in model structure, parametric uncertainty and the inherent variability of the system being modeled, and randomness related to the adequacy of performance under the range of future scenarios the model might be used to simulate [35, 36].

Here, we chose to use a spatially-explicit, individual-based, stochastic model. There are alternative model structures that we could have pursued. Our approach was based on the combined analysis of previous efforts to model interventions against SCFT and the need to address uncertainties faced by modelers while delivering a tool field personnel can adopt for pragmatic decision-making [37]. Spatially-explicit models [38] allow explicit representation of the spatial configuration of different habitats (e.g., a central block of meadows surrounded on three sides by a strip of woods) which affects rates of important system process (e.g. different survival rates of off-host ticks deposited in meadows versus woods). Individual-based models [39, 40] provide a structure that facilitates the investigation of how system-level properties (e.g. the density of host-seeking larvae) emerge from the collective actions of individuals (e.g. non-lactating dairy cattle). Stochastic models [41] allow the explicit representation of parametric uncertainty (e.g. the maximum number of tick larvae allowed on an individual host at any one time) and the inherent variability of the system being modeled (e.g. unpredictable combinations of temperatures, saturation deficits and precipitation).

The modeling experiment reported here provided a framework to illustrate to the stakeholders the need to take an ecological approach for integrated SCFT management in Puerto Rico. This research project allowed us to document that the combined use of safer products with acaricidal properties could reach efficacy levels against SCFT that reduced the risk for morbidity and mortality due to babesiosis and anaplasmosis [18, 19]. Our model could also be used to document the fully integrated use of these acaricidal products with anti-tick vaccine technology as it has been done for example with commonly used acaricides such as organophosphates and pyrethroids and Bm86-based vaccines [20, 42, 43]. Further testing of an experimental Bm86-based vaccine in Puerto Rico will allow for this [44]. Including the cost of different technologies will add value to the model as a tool to document to the producer the return on investment of different intervention modalities. It is expected that helping producers realize financial benefits will facilitate the adoption of cost-effective strategies for integrated SCFT management in the livestock industry of Puerto Rico.

## Conclusions

Explicit simulation of the spatial and temporal dynamics of off-host (host-seeking) SCFT larvae in response to control methods should be an essential element in the evaluation of tick management technologies. Models could also be used to test the cost-effectiveness of integrated SCFT strategies. Following the completion of this research project Puerto Rico was devastated by two hurricanes in 2017 with heavy collateral losses to both the dairy and beef industries. The model presented here is a tool that could be adapted to the current situation to continue efforts towards the integrated use of newer technologies for the sustainable control of SCFT infesting cattle in this Caribbean island.

## Additional files

**Additional file 1.** A detailed model description following the ODD (Overview, Design concepts, and Details) protocol suggested for individual-based models by Grimm et al. [26].

**Additional file 2.** Summary of model parameters and equations.

## Abbreviations

SCFT: Southern cattle fever tick
USDA-ARS: United States Department of Agriculture, Agricultural Research Service
APHIS-VS: Animal Plant Health and Inspection Service-Veterinary Services
ODD: Overview, Design concepts, and Details
SD: Standard deviation
