# Supplementary material for: Simulation tools for assessment of tick suppression treatments of Rhipicephalus (Boophilus) microplus on non-lactating dairy cattle in Puerto Rico
Source: Parasit Vectors. 2019 Apr 27;12:185. doi: 10.1186/s13071-019-3443-6 (PMC6487003; doi:10.1186/s13071-019-3443-6)
Supplement: Supplementary file 1 — Additional file 1. A detailed model description following the ODD (Overview, Design concepts, and Details) protocol suggested for individual-based models by Grimm et al. [26]. [file 13071_2019_3443_MOESM1_ESM.docx]

**Additional file 1**

**Overview**

This appendix contains a detailed model description following the ODD (Overview, Design concepts, and Details) protocol [[1](#_ENREF_1)] A summary of model parameter values and equations is provided in Additional file 2.

**Model description**

**Purpose**

The purpose of the model is to examine the relationship between the proportion of the non-lactating dairy cows treated with acaricides and the efficacy of control of southern cattle ticks in Puerto Rico.

**Entities, state variables, and scales**

Entities include (1) 900, square, 1-ha habitat cells arrayed in a 30 x 30 matrix and (2) a specified number of individual cattle (hosts of cattle fever ticks). State variables, or attributes, of habitat cells include: location (x and y coordinates), habitat type (meadows or woods), and current numbers of tick eggs, larvae, and engorged (fed) adults in each weekly age-class located in the cell, as well as the current numbers of cattle located in the cell. Attributes of cattle include location of the center of their activity range (x and y coordinates), current location (x and y coordinates), habitat type of current location (meadows or woods), relative habitat preferences (a value between 0 (low preference) and 1 (high preference) for each habitat type), size of activity range (ha), and numbers of larval, nymphal, and adult ticks in each age-class they currently are carrying (on-host ticks advance through 30 age classes each week). Global variables representing environmental conditions, which are updated weekly over a 5-year period, include temperature (^o^C), saturation deficit (MB), and a precipitation index (cm).

The determination of temporal and spatial scales was based on the ecology of the organisms involved, the level of detail contained in available information, and computational considerations. Weekly time steps allow adequate representation of the effects of temperature, saturation deficit, and precipitation on the various off-host stages of the tick life cycle [[2](#_ENREF_2)][. Thirty time steps per week provide adequate representation of the spatial-temporal redistribution of ticks which result from the 30 movements per week of each host about the landscape (see Section of “Update attributes of landscape cells representing number of ticks in each off-host developmental phase”).](#_bookmark54)  A 5-year simulation period provides adequate time for both the abundance and distribution of ticks to respond to the tick control methods simulated in the model. A spatial resolution of 1 ha provides adequate detail to represent model processes and to present model results, and also is consistent with the general information available for landscape classiﬁcation [[3](#_ENREF_3)] and the description of cattle habitat use [[4](#_ENREF_4), [5](#_ENREF_5)]. The spatial extent represented by 900 1-ha habitat cells arrayed in a 30 x 30 matrix is adequate to describe landscape structure and cattle movements typical of Puerto Rico and to demonstrate model application under the particular management scenarios considered in this paper.

**Process overview and scheduling**

The model was programmed and simulations were executed in NetLogo (<http://ccl.northwestern.edu/netlogo/>) [[6](#_ENREF_6)]. Simulation results were exported by NetLogo as text files, which were imported by Excel© ([Microsoft,](#_bookmark49) [2013)](#_bookmark49) for archiving, statistical analyses, and temporal graphics. During each simulation, the NetLogo program ﬁrst initializes the system by creating and assigning attributes to the landscape cells and the individual hosts ([Fig. S1)](#_bookmark10). The program then reads input data in the form of time series of values representing the climatic conditions under which the simulation will be run. Next, the program iteratively executes the six submodels, which are nested in two loops. At the beginning of the first time step of each simulated week, the program (1) updates climatic conditions and recalculates associated off-host tick survival and development rates and (2) updates attributes of landscape cells representing the number of ticks in each off-host developmental phase. Then, 30 times during the week, the program iteratively executes submodels 3 through 5. The order in which individual cattle are selected to perform the activities within each submodel is randomized at the beginning of each execution of the submodel. During each iteration, the program first (3) calculates the number of off-host larvae collected by each host and updates the corresponding attributes of landscape cells (off-host larvae) and individual hosts (on-host larvae). Then it (4) calculates the survival and development of on-host ticks (larvae, nymphs, and adults), calculates the number of engorged female ticks deposited by each host in each landscape cell, and updates the corresponding attributes of landscape cells (engorged female ticks) and individual hosts (on-host larvae, nymphs, and adults). Then it (5) moves each host to a new landscape cell within their activity range and updates the corresponding attributes of landscape cells (number of hosts) and individual hosts (current location and habitat type). Finally, at the end of the last time step of each simulated week, the program (6) writes output to text files, which includes current values of climatic conditions, landscape attributes, and host attributes, as well as system-level statistics (e.g., mean (SD), maximum and minimum values) summarizing landscape attributes and host attributes.

**Design concepts**

Basic principles

The biological concept underlying the model design is that the CFT life cycle is influenced by climatic conditions, landscape structure, and availability of hosts. Survival and development of off-host stages are dependent on the temperature and relative humidity in habitat-types to which they are exposed as a consequence of host-driven dispersal of gravid females. Completion of the tick life cycle depends on larvae encountering an appropriate host, which is influenced by species abundance and habitat preferences of hosts. With the goal of providing a useful tool for exploring potential efficacy of different management schemes for controlling CFT on cattle, the model has been designed to represent explicitly the effects of climatic variability, landscape heterogeneity, and host availability on tick population dynamics. Climatic variability is represented via input data including time series of historical data representing weekly temperatures, saturation deficits, and precipitation. Landscape heterogeneity is represented via creation of a spatially-explicit hypothetical landscape consisting of two different habitat types. Availability of hosts is represented via creation of individuals that move about the landscape, collecting and depositing ticks. Individual hosts enter and are removed from the system during each simulation such that the availability of hosts varies through time.

Emergence

Spatial and temporal patterns of abundance off-host tick larvae emerge as system-level properties as a result of equations describing rates of off-host tick development and survival, and rules governing the movements of hosts within the landscape. Rates of off-host tick development and survival depend on the temporally-variable climatic conditions (temperature, saturation deficit, precipitation). Movements of hosts within the landscape depend on the spatially-heterogeneous distribution of habitat types, with probabilistic movement rules favoring selection of landscape cells with the habitat types more preferred by the host. The mean number of adult ticks per individual host also emerges as system-level property. However, the general temporal patterns associated with these means can be anticipated based on the temperature-dependent seasonal variation in the activity level of off-host (host-seeking) tick larvae, the fixed survival rates of ticks on hosts, and the fixed maximum limits on the number of larvae allowed on hosts.

Adaptation

Individual hosts do not possess adaptive traits, the rules for their behavior (movement and collection and deposition of ticks) are fixed.

Objectives

Individual hosts do not adapt their behavior to achieve specific objectives.

Learning

Individual hosts do not learn, that is, they do not change their behavior as a result of past experience.

Prediction

Individual hosts do not predict the future, that is, they do not estimate future conditions nor judge the consequences of their behavior.

Sensing

Individual landscape cells sense (are “aware” of) their habitat type, which affects the survival rates of off-host ticks located in the cell. Individual hosts sense the location of the center of their activity range, the size of their activity range, their habitat preferences, the proportions of landscape cells of each habitat type within their activity range, and the maximum number of larvae they can carry at any given time. For both landscape cells and individual hosts, the mechanisms by which this sensing occurs is implicit, that is, there are no explicit rules governing the acquisition of this information.

Interaction

Interactions between landscape cells and individual hosts occur implicitly via the collection and dispersal of ticks among the landscape cells within the activity ranges of individual hosts. Interactions among individual hosts occur implicitly in that tick larvae collected by one host cannot be collected by other hosts.

Stochasticity

During initialization of the model, the program probabilistically selects the permanent center of the activity range of each individual host based on the relative habitat preferences of the host, and places the host in that center habitat cell. During simulations, the next habitat cell to which a host moves is selected probabilistically based on the relative habitat preferences of the host. This stochasticity results in a probabilistic distribution of hosts available to off-host tick larvae (host-seeking larvae), and a probabilistic distribution of engorged female ticks deposited by hosts. Thus, stochasticity within the model causes host activities to occur with specified mean frequencies within the constraints imposed by the general structure of the landscape.

Collectives

There are no collectives in the model.

Observation

The model records weekly values for the number of eggs, larvae, and engorged adults in each habitat cell, and the number of ticks in each life stage on each host. These data are used primarily for verifying that model code is functioning appropriately. Summary output data collected from the model include the system-level densities of host-seeking larvae, the mean number of adult ticks on cattle.

**Initialization**

The program creates a hypothetical landscape typical of an area used for pasturing the non-lactating pre-parturient cows and heifers within a milking herd operation in Puerto Rico, which for this scenario consists of 70% meadows in forage production and 30% woods distributed around the perimeter of the pasture. Thus the hypothetical landscape for each simulation consists of 630 ha of meadows and 270 ha of woods. The spatial distribution of these habitat types across the landscape does not change during a simulation run or from one simulation run to another simulation, and consists of a central block of meadows surrounded on three sides by a strip of woods which is 90 m wide. Each grid cell initially is assigned 100,000 larvae.

The program creates 63 individual cattle (0.7 individuals per hectare) to represent tick hosts. Based on producer surveys, this represents a typical density of non-lactating dairy cows within a milking herd operation in Puerto Rico. Cattle are assigned an activity range of 300 ha [[7](#_ENREF_7)] relative habitat use preferences for meadows and woods of 0.7 and 0.3, respectively, and an index controlling the maximum number of larvae they can carry at any given time is set at 100 (calibration parameter). Each host is assigned 0 on-host ticks (0 larvae, 0 nymphs, 0 adults). The number of cattle remains constant during each simulation, as do sizes of activity ranges and habitat use preferences. Each host is placed in a landscape cell that is chosen probabilistically based on its relative habitat use preferences. This initial cell becomes the permanent center of the host’s activity range.

**Input data**

The program reads three, 5-year time series (1994-1998) of historical data representing (1) weekly temperatures (^o^C), (2) weekly saturation deficits (MB), and (3) weekly values of an index based on precipitation (cm) for the southwestern portion of Puerto Rico (Fig. S2). These data were obtained from the Southern Regional Climate Center, Baton Rouge, Louisiana, USA.

**Submodels**

Execute tick control if applicable

At the beginning of each week, the program checks to see if tick control should be applied and, if so, executes the tick control method being simulated. Tick control methods include the application of acaricides at a specified frequency (number of weeks between applications) for a specified duration (number of consecutive months) to a specified percentage of the cattle (100, 90, 80, etc.). The effect of acaricides is represented by assigning to each treated cow an acaricide-induced mortality factor which represents the current efficacy of the acaricide on that individual. Acaricide efficacy is assumed to be 90% during the first week after application (that is, it will kill 90% of the on-host ticks during the first week), 95% during each of the next three weeks, and then decrease weekly to 85%, 75%, 65%, %55, 35%, and 0 during the following six weeks. The acaricide-induced mortality is added to the natural mortalities of on-host larval, nymphal, and adult ticks. The assessment criteria include (1) whether or not host-seeking larvae were eliminated from the system and, if so, (2) the number of weeks after the first acaricide application to elimination.

Update climatic conditions and recalculate associated off-host tick survival and development rates

At the beginning of each week, the program updates temperature, saturation deficit, and the precipitation index based on values in the corresponding time series of inputs (Section [of “Input data”).](#_bookmark14) Based on these new environmental conditions, the program recalculates off-host survival and development rates for tick eggs, larvae, and engorged adults in each type of habitat, as well as the relative rate of host-seeking activity of off-host larvae, based on information presented by Mount et al. [[2](#_ENREF_2)]. The program also calculates the number of eggs laid per ovipositing female as a function of temperature [[2](#_ENREF_2)]. The program calculates development rates of eggs and engorged (fed, after blood meal) adults as functions of cumulative “degree-weeks” of temperature; survival rates of eggs and off-host engorged adults as functions of current (weekly) temperature, saturation deficit, and precipitation, as modified by habitat type; and relative rates of host-seeking activity of off-host larvae (the proportion of off-host larvae that potentially could encounter and attach to a host) as functions of current (weekly) temperature following Mount et al. [[2](#_ENREF_2)].

Update attributes of landscape cells representing number of ticks in each off-host developmental phase

At the beginning of each week, the program updates each landscape cell in terms of (1) the number of tick eggs, larvae, and engorged adults that have survived, (2) the number of eggs that have developed into larvae, (3) the number of engorged (female) adults that have laid eggs and died, and (4) the number of eggs laid. The survival of eggs, larvae, and engorged adults depends on temperature, saturation deficit, and the precipitation index, as well as the habitat type of the landscape cell following Mount et al. [[2](#_ENREF_2)]. The development of eggs and engorged adults depends on temperature following Mount et al. [[2](#_ENREF_2)].

Calculate number of off-host larvae collected by hosts and update corresponding attributes of landscape cells and individual hosts

Thirty times during each week, preceding each of the 30 rounds of host movements (see Section of “Move hosts within the landscape and update corresponding attributes of landscape cells and individual hosts” below), the program calculates the number of off-host larvae that are collected by each host from each landscape cell and updates the number of larvae in each cell and the number of larvae on each host. Each individual host has a probability of collecting larvae that depends upon the abundance and relative activity level of larvae in that cell, with an upper limit on the number of larvae that can be collected determined by the number of larvae already on the host. The relative activity level of off-host larvae depends on temperature following Mount et al. [[2](#_ENREF_2)] and the maximum number of larvae allowed on a host is a calibration parameter (see Section of “Model calibration, sensitivity analysis, and evaluation” in the main text).

Calculate survival and development of on-host ticks, calculate number of on-host ticks deposited by hosts, and update corresponding attributes of landscape cells and individual hosts

Thirty times during each week, preceding each of the 30 rounds of host movements (see Section of “Move hosts within the landscape and update corresponding attributes of landscape cells and individual hosts” below) and following the collection of off-host larvae during each round (see Section of “Calculate number of off-host larvae collected by hosts and update corresponding attributes of landscape cells and individual hosts” above), the program calculates the survival and development of the larval, nymphal, and adult ticks on each host, and updates the corresponding attributes of each host. The duration of on-host life stages is one week for larvae, one week for nymphs, and one to two weeks for adults, following Mount et al. [[2](#_ENREF_2)], and the on-host survival rates for larvae, nymphs, and adults also follow Mount et al. [[2](#_ENREF_2)]. The program then deposits 10% of the adult ticks that have been on a host for at least three weeks into the landscape cell in which the host currently is located and updates the corresponding attributes of the host and the landscape cell. Thus members of any given cohort of on-host adult ticks are deposited as engorged adults into up to 30 different landscape cells during the 30 host movements that occur during the last week that the adults are on the host.

Move hosts within the landscape and update corresponding attributes of landscape cells and individual hosts

Thirty times during each week, following the collection, on-host survival and development, and deposition of ticks during each of the 30 rounds, the program moves each host within the landscape. The landscape cells visited are selected probabilistically from those within the activity range of the host based on the habitat preferences of the host and the proportion of the different habitat types within the activity range. The program code that executes host movements is provided in the Addition file 2.

Write output summarizing landscape attributes, host attributes, and system-level statistics

At the end of each week, the program writes to text files summaries of climatic conditions (temperature, saturation deficit, precipitation index), selected attributes of landscape cells (density of off-host larvae), hosts (number of on-host adult ticks), and system-level statistics (mean density of off-host larvae in the system, mean number of on-host adult ticks per individual host).

**References**

1. Grimm V, Berger U, DeAngelis DL, Polhill JG, Giske J, Railsback SF. The ODD protocol: a review and first update. Ecological Modelling. 2010;221 23:2760-8.

2. Mount G, Haile D, Davey R, Cooksey L. Computer simulation of *Boophilus* cattle tick (Acari: Ixodidae) population dynamics. Journal of Medical Entomology. 1991;28 2:223-40.

3. Program USGSGA: GAP/LANDFIRE National Terrestrial Ecosystems 2011. Edited by Survey USG. Boise, ID2016.

4. Cooper SM, Perotto-Baldivieso HL, Owens MK, Meek MG, Figueroa-Pagan M. Distribution and interaction of white-tailed deer and cattle in a semi-arid grazing system. Agriculture, Ecosystems & Environment. 2008;127 1-2:85-92.

5. Cohen WE, Drawe DL, Bryant FC, Bradley LC. Observations on white-tailed deer and habitat response to livestock grazing in south Texas. Journal of Range Management. 1989:361-5.

6. Wilensky U: NetLogo. <http://ccl.northwestern.edu/netlogo/>. Evanston, IL: Center for Connected Learning and Computer-Based Modeling, Northwestern University; 1999.

7. Howery LD, Provenza FD, Banner RE, Scott CB. Differences in home range and habitat use among individuals in a cattle herd. Applied Animal Behaviour Science. 1996;49 3:305-20.

**
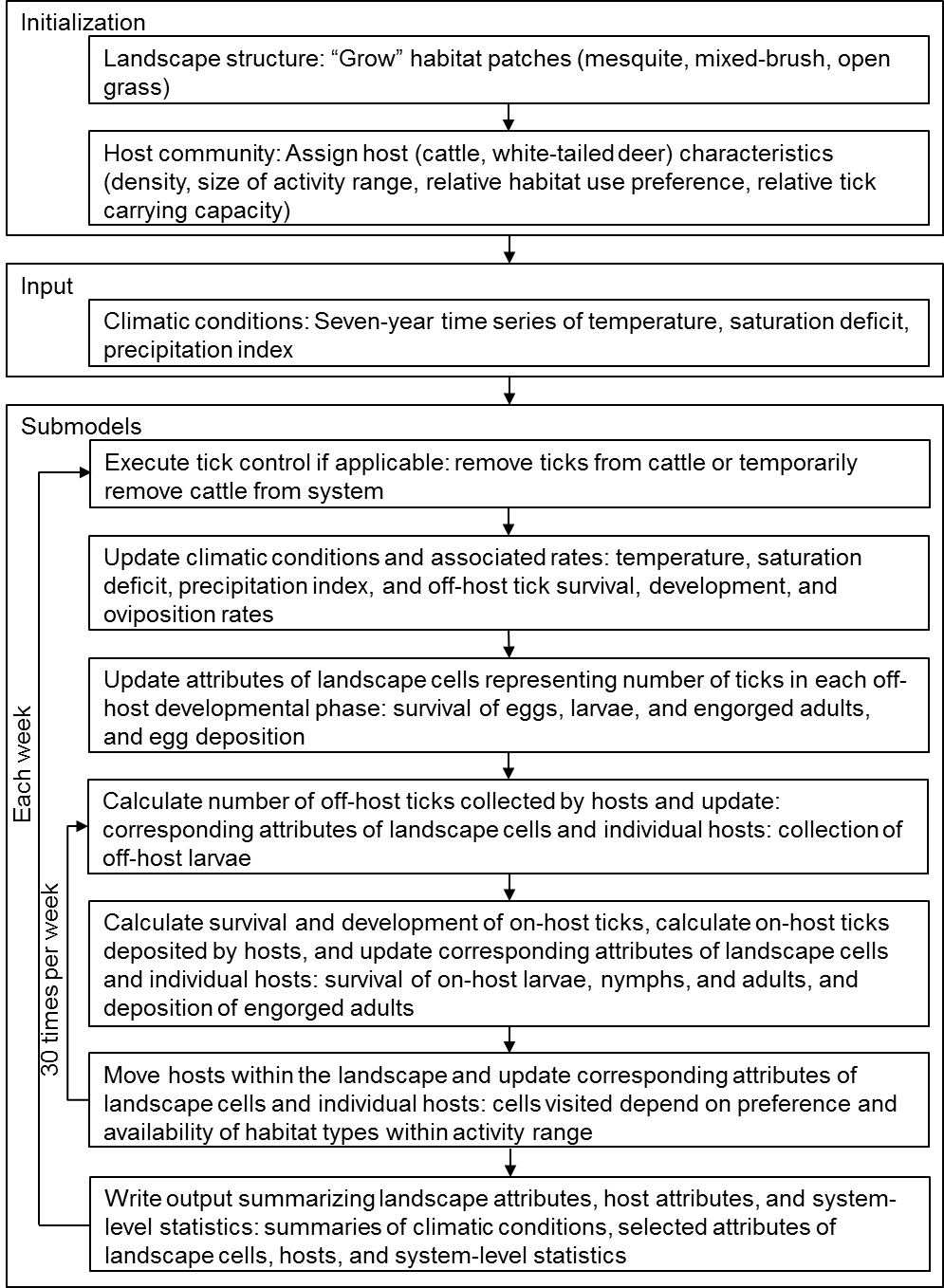
**

Figure S1. Overview of the sequence of events and processes involved in the execution of the model to simulate population responses of the southern cattle tick *Rhipicephalus* (*Boophilus*) *microplus* to integrated tick management in Puerto Rico.


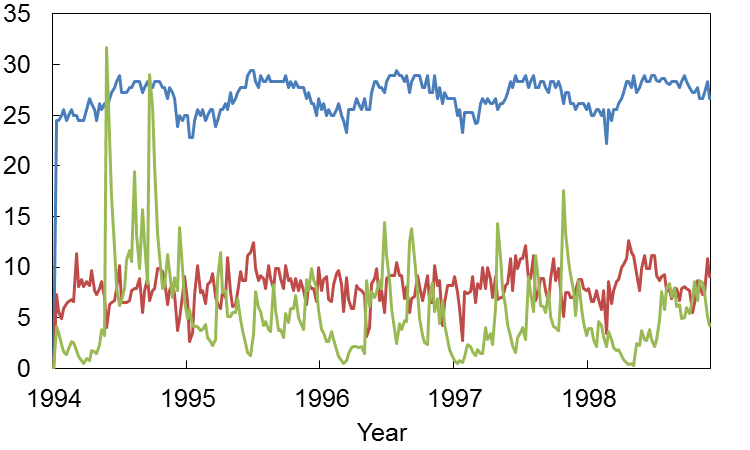


Figure S2. Five-year time series (1994-1998) of historical data used during simulations to represent weekly temperatures (^o^C, blue line), weekly saturation deficits (MB, red line), and weekly values of an index based on precipitation (cm, green line) for the southwestern portion of Puerto Rico. These data were obtained from the Southern Regional Climate Center, Baton Rouge, Louisiana, USA.
