# Supplementary material for: Simulation tools for assessment of tick suppression treatments of Rhipicephalus (Boophilus) microplus on non-lactating dairy cattle in Puerto Rico
Source: Parasit Vectors. 2019 Apr 27;12:185. doi: 10.1186/s13071-019-3443-6 (PMC6487003; doi:10.1186/s13071-019-3443-6)
Supplement: Supplementary file 2 — Additional file 2. Summary of model parameters and equations. [file 13071_2019_3443_MOESM2_ESM.docx]

**Addition file 2: Summary of model parameters and equations**

Parameters and equations are based on information presented in Mount et al. [[1](#_ENREF_1)] unless otherwise specified.

**Climatic variables**

Mean temperature (T)

Saturation deficit (SD); $SD=SVP\times\left( 1-RH \right)/100$ where *SVP* is the saturation vapor pressure and *RH* is the relative humidity

Precipitation index (PI); $PI_{t}=P_{t}+{PI}_{t-1}\times\left( 1-L \right)$ where *P_t_* is the precipitation in week *t* and L is a weekly moisture loss rate and a constant of 0.35 equaling about 99% loss in the index over 10 weeks period.

**Fecundity (*F*; eggs/engorged female)**

Depend on the mean temperature (*T*; 12̊C-32̊C) during preoviposition

$F=-7487.5+790\cdot T-14.5{\cdot T}^{2}$; for ticks obtaining blood meal from cattle

**Survival of eggs (proportion/week)**

Base survival rates (*S_E_,* under optimal weather conditions) in three different types of pasture

CDW (cumulative degree-weeks) >=36

DT (minimum developmental threshold temperature) = 15

1-4 weeks old, $S_{E1}=\left\{ \begin{matrix} 0.964264 (improved dense pasture) \\ 0.9362846 (unimproved light pasture) \\ 0.888922 (improved light) \end{matrix} \right.$

> 4 weeks old, $S_{E2}=\left\{ \begin{matrix} 0.8344503 (improved dense pasture) \\ 0.8015637 (unimproved light pasture) \\ 0.7148943 (improved light) \end{matrix} \right.$

Note that for habitat-specific tick survival parameters, we have retained the original habitat names in Mount et al. [[1](#_ENREF_1)]: “improved dense pasture,” “unimproved light pasture,” and “improved light,” which they used to refer to good, fair, and poor habitats, respectively, relative to survival of off-host ticks. These parameter values were based on maximum survival times reported in eight independent studies conducted in a variety of habitats (see page 225 in Mount et al. [[1](#_ENREF_1)]). In the text describing our study, we have used the names “woods” and “meadows” to refer to good and poor habitats, respectively, relative to survival of off-host ticks.

Survival rate effects (*SRE*) for the temperature (*T*), saturation deficit (*SD*), and precipitation index (*PI*) (the following equation, *SRE_E1_* will be used for 1-4 weeks old eggs, 1-6 weeks old larvae, and 1-4 weeks engorged females, and *SRE_old_* will be used for > 4 weeks old eggs, > 6 weeks old larvae, and > 4 weeks engorged female as well)

1-4 weeks old, ${SRE}_{young}=\left( -0.00097222\cdot T^{2}+0.03111111\cdot T+0.75111111 \right)\times\left( -0.00123643\cdot{SD}^{2}+0.01456543\cdot SD+0.95708835 \right)\times\left( -0.00060557\cdot{PI}^{2}+0.0159513\cdot PI+0.89479707 \right)$

> 4 weeks old, ${SRE}_{old}=\left( -0.00114583\cdot T^{2}+0.036250\cdot T+0.71333333 \right)\times\left( -0.00141020\cdot{SD}^{2}+0.01827455\cdot SD+0.94021589 \right)\times\left( -0.00074267\cdot{PI}^{2}+0.0194957\cdot PI+0.87183511 \right)$

**Survival of free-living (host seeking) larvae (proportion/week)**

Base survival rates (*S_FL_,* under optimal weather conditions) in three different types of pasture

1-6 weeks old, $S_{FL1}=\left\{ \begin{matrix} 0.9539088 (improved dense pasture) \\ 0.9342157 (unimproved light pasture) \\ 0.9001101 (improved light) \end{matrix} \right.$

> 6 weeks old, $S_{FL2}=\left\{ \begin{matrix} 0.9154279 (improved dense pasture) \\ 0.8982628 (unimproved light pasture) \\ 0.8516867 (improved light) \end{matrix} \right.$

Survival rate effects (*SRE*) for the temperature (*T*), saturation deficit (*SD*), and precipitation index (*PI*)

1-6 weeks old, ${SRE}_{young}=\left( -0.00097222\cdot T^{2}+0.03111111\cdot T+0.75111111 \right)\times\left( -0.00123643\cdot{SD}^{2}+0.01456543\cdot SD+0.95708835 \right)\times\left( -0.00060557\cdot{PI}^{2}+0.0159513\cdot PI+0.89479707 \right)$

> 6 weeks old, ${SRE}_{old}=\left( -0.00114583\cdot T^{2}+0.036250\cdot T+0.71333333 \right)\times\left( -0.00141020\cdot{SD}^{2}+0.01827455\cdot SD+0.94021589 \right)\times\left( -0.00074267\cdot{PI}^{2}+0.0194957\cdot PI+0.87183511 \right)$

**Host-finding rate**

Relative rates of host-seeking activity of off-host larvae (*HFR*, the proportion of off-host larvae that potentially could encounter and attach to a host)

$$HFR=-0.008\times T^{2}+0.4\times T-4$$

**Survival of on-host larvae (density-dependent survival on cattle) (proportion/week)**

Survival rate (*S_L_*); depends on cattle type and tick density (ticks per host, *D*)

When tick density (tick exposure index) < 1000, $S_{L}=0.37$;

When 1000 <= tick density (tick exposure index) < 15000, $S_{L}=-0.000013\cdot D+0.382857$;

When 15000 <= tick density (tick exposure index), $S_{L}=0.19$

**Survival of on-host nymphs (density-dependent survival on cattle) (proportion/week)**

Survival rate (*S_N_*); depends on cattle type and tick density (ticks per host, *D*)

When tick density (tick exposure index) < 1000, $S_{N}=0.97$;

When 1000 <= tick density (tick exposure index) < 15000, $S_{N}=-0.000027\cdot D+0.997143$;

When 15000 <= tick density (tick exposure index), $S_{N}=0.59$

**Survival of on-host adults (density-dependent survival on cattle) (proportion/week)**

Survival rate (*S_F_* and *S_M_*); depends on cattle type and tick density (ticks per host, *D*)

Male and female ticks are separated during the transition of nymphs to adults on host using a constant female/male ratio of 1.36:1.00.

Male ticks are contained in one accumulator with no age structure.

The time on host is 1 or 2 weeks for females (50% split).

When tick density (tick exposure index) < 1000, $S_{F}=S_{M}=0.79$;

When 1000 <= tick density (tick exposure index) < 15000, $S_{F}=S_{M}=-0.000023\cdot D+0.812857$;

When 15000 <= tick density (tick exposure index), $S_{F}=S_{M}=0.47$

**Survival of engorged adult females (proportion/week)**

Base survival rates (*S_EF_*, under optimal weather conditions) in three different types of pasture

*CDW* (cumulative degree-weeks) >= 30

*DT* (minimum developmental threshold temperature) = 15

1-4 weeks old, $S_{EF1}=\left\{ \begin{matrix} 0.974004 (improved dense pasture) \\ 0.945742 (unimproved light pasture) \\ 0.897901 (improved light) \end{matrix} \right.$

> 4 weeks old, $S_{EF2}=\left\{ \begin{matrix} 0.8428791 (improved dense pasture) \\ 0.8096603 (unimproved light pasture) \\ 0.7221155 (improved light) \end{matrix} \right.$

Survival rate effects (*SRE*) for the temperature (*T*), saturation deficit (*SD*), and precipitation index (*PI*)

1-4 weeks old, ${SRE}_{young}=\left( -0.00097222\cdot T^{2}+0.03111111\cdot T+0.75111111 \right)\times\left( -0.00123643\cdot{SD}^{2}+0.01456543\cdot SD+0.95708835 \right)\times\left( -0.00060557\cdot{PI}^{2}+0.0159513\cdot PI+0.89479707 \right)$

> 4 weeks old, ${SRE}_{old}=\left( -0.00114583\cdot T^{2}+0.036250\cdot T+0.71333333 \right)\times\left( -0.00141020\cdot{SD}^{2}+0.01827455\cdot SD+0.94021589 \right)\times\left( -0.00074267\cdot{PI}^{2}+0.0194957\cdot PI+0.87183511 \right)$

**Acaricide-induced mortality (additive to natural on-host mortality) (proportion/week)**

The program code that assigns current acaricide efficacy to treated (“dipped”) individual hosts, which is written in NetLogo (<http://ccl.northwestern.edu/netlogo/>) [[2](#_ENREF_2)], is provided below.

to update-acaricide-efficacy

if dipped? = "yes"

[

set last-dip last-dip + 1

; adjusts weekly acaricide mortality rate to account for loss of efficacy since last treatment

if last-dip = 1 [set acaricide-efficacy 0.90]

if last-dip = 2 [set acaricide-efficacy 0.95]

if last-dip = 3 [set acaricide-efficacy 0.95]

if last-dip = 4 [set acaricide-efficacy 0.95]

if last-dip = 5 [set acaricide-efficacy 0.85]

if last-dip = 6 [set acaricide-efficacy 0.75]

if last-dip = 7 [set acaricide-efficacy 0.65]

if last-dip = 8 [set acaricide-efficacy 0.55]

if last-dip = 9 [set acaricide-efficacy 0.35]

if last-dip = 10 [set acaricide-efficacy 0]

;if acaricide-efficacy < 0 [set acaricide-efficacy 0]

;type week-of-year type " " type max-acaricide-efficacy type " " print acaricide-efficacy

]

end ; end of “update-acaricide-efficacy"

**Host movements (based on Wang et al. 2016)**

*The program code that executes host movements, which is written in NetLogo (Wilensky 1999,* [*http://ccl.northwestern.edu/netlogo/*](http://ccl.northwestern.edu/netlogo/)*), is provided below.*

to move-cattle

; calculate the proportion of each habitat type within the core area of the individual

let num-core-area-patches count core-area-patches

let num-woods-patches count core-area-patches with [hab-type = "woods"]

let num-meadow-patches count core-area-patches with [hab-type = "meadows"]

let num-mixed-brush-patches count core-area-patches with [hab-type = "mixed-brush"]

let pro-woods-patches num-woods-patches / num-core-area-patches

let pro-meadow-patches num-meadow-patches / num-core-area-patches

let pro-mixed-brush-patches num-mixed-brush-patches / num-core-area-patches

; adjust habitat preferences of the individual if not all habitat types are available within the core area

let adjust-pref-woods pref-woods

let adjust-pref-meadows pref-meadows

let adjust-pref-mixed-brush pref-mixed-brush

ifelse pro-woods-patches > 0 and pro-meadow-patches > 0 and pro-mixed-brush-patches > 0

[

; do not adjust preferences if all habitat types are available within the core area

]

[

if pro-woods-patches = 0 and pro-meadow-patches > 0 and pro-mixed-brush-patches > 0

[

set adjust-pref-woods 0

set adjust-pref-meadows pref-meadows / (pref-meadows + pref-mixed-brush)

set adjust-pref-mixed-brush pref-mixed-brush / (pref-meadows + pref-mixed-brush)

]

if pro-woods-patches > 0 and pro-meadow-patches = 0 and pro-mixed-brush-patches > 0

[

set adjust-pref-woods pref-woods / (pref-woods + pref-mixed-brush)

set adjust-pref-meadows 0

set adjust-pref-mixed-brush pref-mixed-brush / (pref-woods + pref-mixed-brush)

]

if pro-woods-patches > 0 and pro-meadow-patches > 0 and pro-mixed-brush-patches = 0

[

set adjust-pref-woods pref-woods / (pref-woods + pref-meadows)

set adjust-pref-meadows pref-meadows / (pref-woods + pref-meadows)

set adjust-pref-mixed-brush 0

]

if pro-woods-patches = 0 and pro-meadow-patches = 0 and pro-mixed-brush-patches > 0

[

set adjust-pref-woods 0

set adjust-pref-meadows 0

set adjust-pref-mixed-brush 1

]

if pro-woods-patches > 0 and pro-meadow-patches = 0 and pro-mixed-brush-patches = 0

[

set adjust-pref-woods 1

set adjust-pref-meadows 0

set adjust-pref-mixed-brush 0

]

if pro-woods-patches = 0 and pro-meadow-patches > 0 and pro-mixed-brush-patches = 0

[

set adjust-pref-woods 0

set adjust-pref-meadows 1

set adjust-pref-mixed-brush 0

]

]

; move individual

let ran-move random-float 1

if ran-move < adjust-pref-woods [move-to one-of core-area-patches with [hab-type = "woods"] set in-hab [hab-type] of patch-here]

if ran-move >= adjust-pref-woods and ran-move < adjust-pref-woods + adjust-pref-meadows [move-to one-of core-area-patches with [hab-type = "meadows"] set in-hab [hab-type] of patch-here]

if ran-move >= adjust-pref-woods + adjust-pref-meadows [move-to one-of core-area-patches with [hab-type = "mixed-brush"] set in-hab [hab-type] of patch-here]

;ask patch-here [set pcolor yellow]

if species = 1 [ask patch-here [set cattle-use cattle-use + 1 set cattle-use-today cattle-use-today + 1]]

if species = 2 [ask patch-here [set deer-use deer-use + 1 set deer-use-today deer-use-today + 1]]

if species = 3 [ask patch-here [set nilgai-use nilgai-use + 1 set nilgai-use-today nilgai-use-today + 1]]

; record time spent in this habitat type

if in-hab = "woods" [set time-in-woods time-in-woods + 1]

if in-hab = "meadows" [set time-in-meadows time-in-meadows + 1]

if in-hab = "mixed-brush" [set time-in-mixed-brush time-in-mixed-brush + 1]

end ; end of "move-cattle";

Reference

1. Mount G, Haile D, Davey R, Cooksey L. Computer simulation of *Boophilus* cattle tick (Acari: Ixodidae) population dynamics. Journal of Medical Entomology. 1991;28 2:223-40.

2. Wilensky U: NetLogo. <http://ccl.northwestern.edu/netlogo/>. Evanston, IL: Center for Connected Learning and Computer-Based Modeling, Northwestern University; 1999.
